# Poor adherence and low persistency rates for hepatocellular carcinoma surveillance in patients with chronic hepatitis B

**DOI:** 10.1097/MD.0000000000004744

**Published:** 2016-09-02

**Authors:** Christina Wang, Vincent Chen, Vinh Vu, An Le, Linda Nguyen, Changqing Zhao, Carrie R. Wong, Nghia Nguyen, Jiayi Li, Jian Zhang, Huy Trinh, Mindie H. Nguyen

**Affiliations:** a Public Policy Department, Stanford University, Stanford, CA; b Department of Medicine, Stanford University Medical Center, Palo Alto, CA; c Division of Gastroenterology and Hepatology, Stanford University Medical Center, Palo Alto, CA; d Department of Biology, Stanford University, Stanford, CA; e Department of Cirrhosis, Institute of Liver Disease, Shuguang Hospital, Shanghai University of T.C.M., Shanghai, P.R. China; f Department of Medicine, Yale University Medical Center, New Haven, CT; g Department of Medicine, University of California, San Diego Medical Center, San Diego, CA; h Department of Gastroenterology, Palo Alto Medical Foundation, Palo Alto, CA; i Department of Outpatient Clinics, Chinese Hospital, San Francisco, CA; j San Jose Gastroenterology, San Jose, CA.

**Keywords:** cirrhosis, compliance/adherence, hepatocellular carcinoma, screening

## Abstract

Our goal was to examine rates and predictors for hepatocellular carcinoma (HCC) surveillance adherence and persistency, since studies of such adherence and persistency in patients with chronic hepatitis (CHB) are currently limited.

Consecutive CHB patients (N = 1329) monitored for ≥1 year at 4 US clinics from January 1996 to July 2013 were retrospectively studied. Surveillance adherence was evaluated based on the American Association for the Study of Liver Diseases guidelines. Kaplan–Meier method was used to analyze surveillance persistency of 510 patients who had initially fair adherence (having at least annual surveillance imaging with further follow-up).

Mean age was 48, with the majority being male (58%), Asian (92%), foreign-born (95%), and medically insured (97%). Patients with cirrhosis and those seen at university liver clinics were more likely to have optimal HCC surveillance than those without cirrhosis and those seen at community clinics (38.4% vs 21.6%, *P* <0.001 and 33.5% vs 14.4%, *P* < 0.001, respectively). HCC diagnosed in optimally adherent patients trended toward smaller tumor size (*P* < 0.08). On multivariate analysis also inclusive of age, sex, clinical visits, cirrhosis, clinic setting and antiviral therapy use, strong independent predictors for having at least annual imaging were a history of more frequent clinical visits (odds ratio [OR] = 2.5, *P* < 0.001) and university-based care (OR = 5.2, *P* < 0.001). Even for those with initially fair adherence, persistency dropped to 70% at 5 years.

Adherence and persistency to HCC surveillance in CHB patients is generally poor. More frequent clinic visits and university-based settings were significant and strong predictors of at least annual HCC surveillance adherence.

## 1 Introduction

Hepatocellular carcinoma is the fifth most commonly diagnosed cancer and second leading cause of cancer-related death in men, and the seventh most commonly diagnosed cancer and sixth leading cause of cancer-related death in women worldwide.^[1,2]^ Established risk factors for hepatocellular carcinoma (HCC) include cirrhosis of any etiology and infection with hepatitis B virus (HBV) with or without cirrhosis.^[3,4]^

Survival for HCC patients is very low with 5-year relative survival rates of only 5% from 1987 to 1989, which modestly improved to 18% from 2005 to 2011 in the United States.^[5]^ Since HCC is typically asymptomatic in its earliest stage, the only hope of early detection in the presymptomatic stage lies in routine screening and surveillance.^[6]^ Several studies have shown that early detection of HCC and subsequent curative treatment can lead to improved clinical outcomes,^[6,7]^ and a meta-analysis of 47 studies with a total of 15,158 patients found that HCC surveillance was associated with improved early stage detection (odds ratio [OR] 2.08, 95% confidence interval [CI] 1.80–2.37) as well as significantly prolonged survival (OR 1.90, 95% CI 1.67–2.17).^[8]^ Unfortunately, based on another meta-analysis of 28 studies and 15,244 patients, early asymptomatic HCC accounts for only approximately 30% of patients at initial presentation.^[9]^

Current guidelines by the American Association for the Study of Liver Diseases (AASLD) recommend ultrasound examination at 6-month intervals for chronic hepatitis B (CHB) patients who are Asian males aged 40 or older, Asian females aged 50 or older, those with cirrhosis, African or North American blacks, and those with a family history of HCC.^[10]^ Unfortunately, prior studies in community primary care and gastroenterology (GI) practices, the Veterans Affairs Healthcare System, and Medicaid populations have suggested that adherence to HCC surveillance guidelines is very poor.^[11–15]^

Little is known regarding the variability in HCC surveillance adherence in CHB patients by cirrhosis status. In addition, current studies have largely focused on 1 single center, medical insurance program, 1 type of medical practice, or are based on anonymous survey data rather actual clinical practice data.^[12–17]^ A study of HCC surveillance in a Medicaid cirrhotic population found that only 26% underwent at least 1 imaging test over a 15-month period.^[15]^ Another study of HCC surveillance of 557 patients in community GI clinics found that about 40.6% of the cohort received poor or no surveillance.^[11]^ However, little is known regarding the potential variability in surveillance between academic and community clinics serving patients with liver disease. Lastly, besides initial adherence to HCC surveillance, the persistency of optimal HCC surveillance has particularly been poorly studied.

In this multicenter study we examined the adherence and persistency to adherence to HCC surveillance in a large and diverse population of CHB patients with specific focus on differences by disease status (cirrhosis vs noncirrhosis) and clinical settings (community vs academic practice).

## 2 Materials and methods

### 2.1 Study population

A total of 2643 patients were identified via computer query using International Classification of Disease, Ninth Revision codes (070.32 for chronic hepatitis B) from 1 of 4 US GI, primary care, or liver clinic study centers from the San Francisco Bay Area. Patients were eligible for inclusion if they were diagnosed with CHB and monitored ≥1 year between January 1996 and July 2013, as verified by medical chart review using a case report form.

All CHB diagnoses were verified by chart review upon evidence of positive hepatitis B surface antigen (HBsAg) or positive HBV deoxyribonucleic acid (DNA) tests at least 6 months apart. All cirrhosis diagnoses were verified by chart review based on histologic diagnosis of cirrhosis in addition to mention of any of the following in radiology, laboratory records, or physician's notes: nodular contour, ascites, encephalopathy, splenomegaly, esophageal varices, other varices, or platelets <120,000/mL. Follow-up time was defined as the duration from initial presentation with CHB at study centers to the most recent patient encounter, incident HCC diagnosis, liver transplantation for non-HCC indications, or death.

All imaging and patient clinical data were collected from review of medical records and confirmed through assessing physician orders, laboratory reports, and radiology records. Imaging tests included in the study analysis were performed for surveillance purposes and could have been ordered by any of the patients’ care providers, including primary care physicians, gastroenterologists, and hepatologists; additionally, imaging tests were included as completed surveillance if they were completed by the patient.

A total of 1314 patients were excluded for the following reasons: <12 months of follow-up (N = 1039); HCC diagnosis prior to first visit (N = 76); infection with hepatitis C virus, hepatitis D virus, or human immunodeficiency virus (N = 62); unknown HCC diagnosis date (N = 58); HCC diagnosis within first 12 months of follow-up (N = 33); prior liver transplantation (N = 30); missing HbsAg data (N = 12), and age <18 years (N = 4).

The final retrospective cohort used for analysis included 1329 patients consecutive patients with CHB, with and without cirrhosis, and at least 12 months of follow-up.

### 2.2 Definition and categories of surveillance and persistency

Adherence was classified as optimal if imaging was performed every 6 months, suboptimal if performed only every 6 to 12 months, poor if performed less often than every 12 months, and none if there was no surveillance at all.

For persistency analysis, the Kaplan–Meier method was used to analyze the 510 patients who had either optimal or suboptimal adherence (imaging at least once every 12 months) for at least 1 year and were available for further follow-up. Persistent surveillance was defined as receiving imaging at least once every 12 months and was lost if a patient received an imaging surveillance test greater than 12 months from the last imaging test. Patients were censored if they developed new HCC, died, underwent liver transplantation, or were lost to follow-up.

### 2.3 Statistical analysis

Descriptive statistics were performed using proportions (%) for categorical variables and mean ± standard deviation or median (range) for continuous variables. Comparative analysis between groups by cirrhosis status or clinic setting was performed using the *χ*^*2*^ test for categorical variables and the Student *t* test or rank-sum test for continuous variables depending on whether a normal distribution of values was observed or not. Stepwise multivariate logistic regression was used to estimate odds ratios and 95% CI relating potential predictors to the outcomes of optimal or suboptimal imaging surveillance adherence or persistency. For survival analysis, the Kaplan–Meier method was used to illustrate persistency to adherence to HCC surveillance by cirrhosis status and clinic setting. Comparison of persistency between the groups was evaluated using the log-rank test. All statistical analysis was performed using Stata 13 (Stata Corporation, College Station, TX). This study was approved by the Institutional Review Board at Stanford University, Stanford, CA.

## 3 Results

### 3.1 Patient characteristics by presence of cirrhosis and clinical settings

Table 1 presents the cohort's demographic and clinical characteristics overall and by cirrhosis status. The overall mean age was 48, with the majority of patients being male (58%), Vietnamese or Chinese (92%), foreign-born (95%), and medically insured (97%). The median follow-up after initial visit was 56 months and the median average clinical visits per year was just under 2 (1.88 visits/year). Patients with cirrhosis comprised 12.3% of the total cohort. Patients with cirrhosis were older (mean age 54 vs 47, *P* < 0.001), more likely male (70.1% vs 56.7%, *P* = 0.001), more likely to have comorbidities (56.7% vs 41.2%, *P* < 0.001), and more likely to have a history of antiviral therapy (68.3% vs 42.8%, *P* < 0.001). All patients who had cirrhosis had detectable HBV DNA, and 79.8% had HBV DNA >2000 IU/mL, the threshold for treatment of patients with compensated cirrhosis by earlier AASLD guidelines.^[18,19]^ These patients had higher HCC incidence during follow-up (21.3% vs 0.9%, *P* < 0.001) and had more clinical visits per year (2.6 vs 1.8, *P* < 0.001).

**Table 1:**
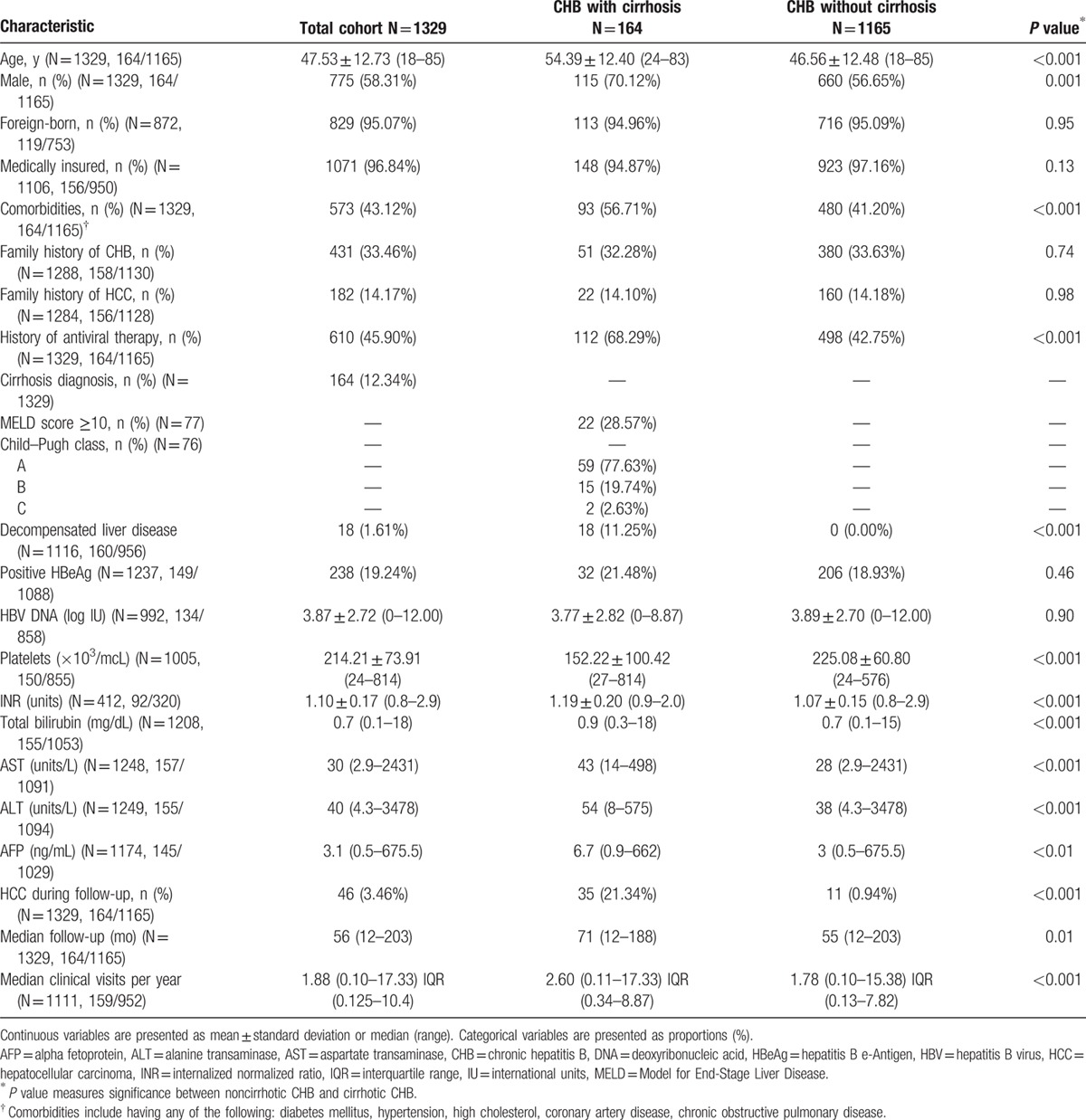
Baseline characteristics, by cirrhosis status.

Table 2 presents the cohort's demographic and clinical characteristics by university or community care settings. Patients seen at university clinics were younger (mean age 43 vs 52, *P* < 0.001), less likely foreign-born (91.2% vs 99.5%, *P* < 0.001), less likely to have comorbidities (36.3% vs 49.6%, *P* < 0.001), more likely to have a family history of HCC (16.6% vs 12.0%, *P* = 0.02), more likely to be diagnosed with cirrhosis (17.8% vs 7.2%, *P* < 0.001), had a longer total period of follow-up (60 vs 54 months, *P* < 0.001), more clinical visits per year (2.0 vs 1.7, *P* < 0.001), and a higher incidence of HCC during follow-up (4.9% vs 2.1%, *P* = 0.004).

**Table 2:**
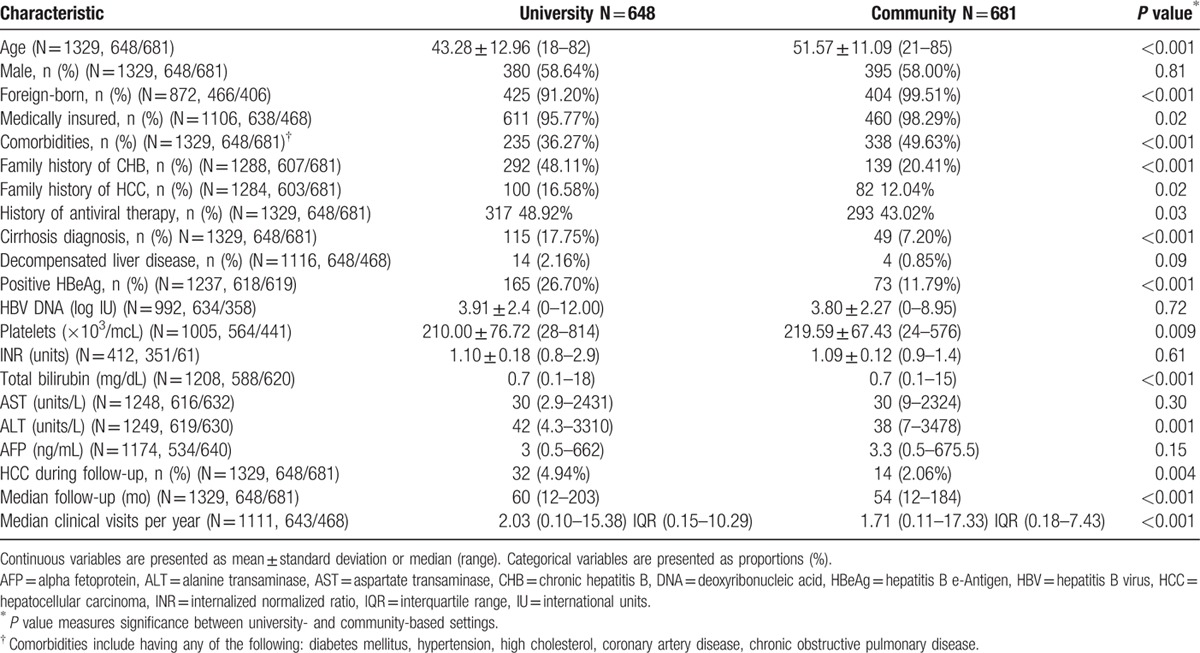
Baseline characteristics of cohort, by practice settings.

### 3.2 Adherence of HCC surveillance rates by presence of cirrhosis and clinical settings

**Figure 1:**
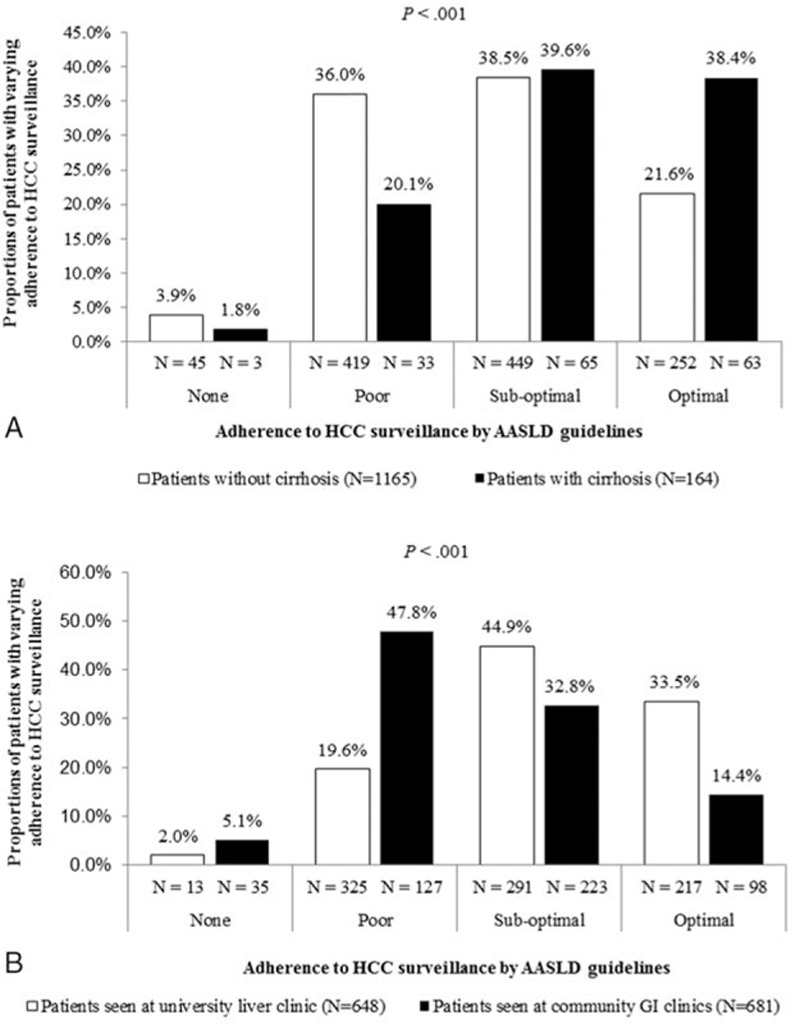
Poor adherence to HCC surveillance in CHB patients regardless of cirrhosis status or clinical setting. A, Adherence to HCC surveillance, by cirrhosis status. B, Adherence to HCC surveillance, by clinical setting. AASLD = American Association for the Study of Liver Diseases, GI = gastroenterology, HCC = hepatocellular carcinoma.

Figure 1A demonstrates the rates of surveillance adherence among patients by cirrhosis status. Approximately 21.9% of patients with cirrhosis and 39.9% of patients without cirrhosis had poor or no surveillance. Patients with cirrhosis were more likely to have optimal HCC surveillance (38.4% vs 21.6%, *P* < 0.001) than those without cirrhosis, and noncirrhotic patients were more likely to have poor or no surveillance. In subanalysis of individuals (1171 of 1329, 88.1%) who only strictly fit the demographics of the AASLD's surveillance guidelines (Asian male hepatitis B carriers older than 40, Asian female hepatitis B carriers older than 50, hepatitis B carrier with a family history of HCC, African/North African blacks with hepatitis B, cirrhotic hepatitis B carriers), adherence rates improved slightly but were still low: 38.4% of those with cirrhosis had optimal surveillance adherence and 23.4% in patients without cirrhosis (*P* < 0.001).

Figure 1B displays the rates of surveillance adherence among patients by clinic setting. Over half (52.9%) of community clinic patients and over one-fifth (21.6%) of university clinic patients received poor to no surveillance overall. Patients seen at university clinics were more likely to have optimal HCC surveillance (33.5% vs 14.4%, *P* < 0.001) than those seen at community clinics, with the latter also more likely to have poor adherence or no surveillance at all. Similarly, in subanalysis of individuals who strictly fit the demographics of the AASLD's surveillance guidelines (1171 of 1329, 88.1%), adherence rates improved slightly but were still low: only 16.0% of patients seen at community clinics received optimal surveillance though a significantly higher proportion (34.2%) of patients seen at university clinics received optimal surveillance (*P* < 0.001).

**Figure 2:**
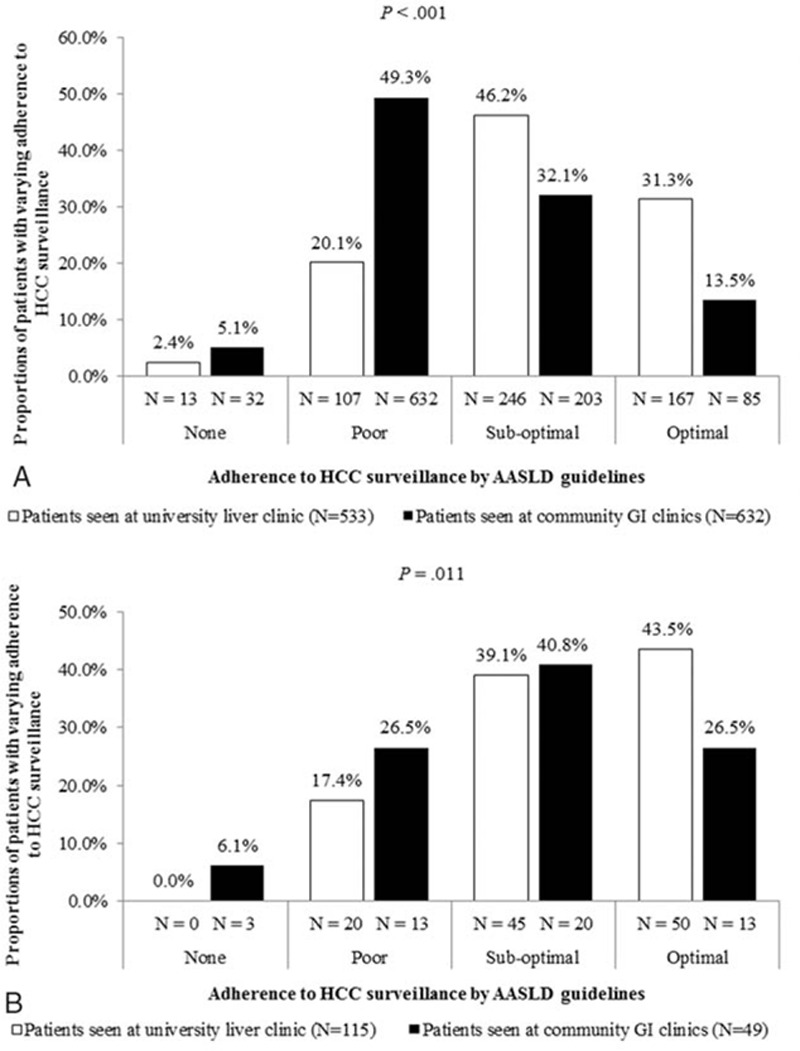
Poor adherence to HCC surveillance in CHB patients when stratified by cirrhosis status, regardless of clinical setting. A, Adherence to HCC surveillance in patients without cirrhosis, by clinical setting. B, Adherence to HCC surveillance in patients with cirrhosis, by clinical setting. AASLD = American Association for the Study of Liver Diseases, GI = gastroenterology, HCC = hepatocellular carcinoma.

Figure 2 compares the rates of surveillance adherence between clinic settings after separating the cohort by cirrhosis status. For patients without cirrhosis, trends of greater optimal surveillance adherence among university patients compared with community clinic patients were observed (31.3% vs 13.5%, *P* < 0.001) (Fig. 2A). Similarly, among patients with cirrhosis, greater optimal surveillance and surveillance adherence among university patients compared with community clinic patients was observed (42.5% vs 26.5%, *P* = 0.011) (Fig. 2B).

### 3.3 Predictors for adherence to HCC surveillance

Table 3 presents predictors for optimal or suboptimal (imaging at least every 6 to 12 months) adherence to surveillance using univariate and multivariate analysis with baseline characteristics including those described in Tables 1 and 2. On multivariate regression also inclusive of age, sex, clinical visits, university-based care (vs community care only), cirrhosis status, and history of antiviral therapy for CHB, strong independent predictors for at least suboptimal imaging adherence were more clinical visits per year (OR = 2.5, *P* <0.001) and university-based care (OR = 5.2, *P* < 0.001). We also examined the effect of HBeAg status, HBV DNA and alanine transaminase levels, insurance status, and presence of comorbidities on univariate analysis but none of these were significant predictors of improved adherence.

**Table 3:**
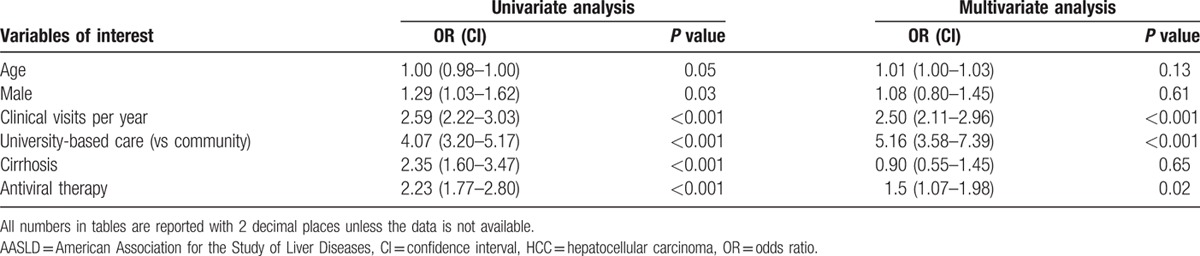
Predictors of optimal or suboptimal hepatocellular carcinoma surveillance adherence to American Association for the Study of Liver Diseases guidelines.

### 3.4 Characteristics of HCC in adherent versus nonadherent patients

A total of 46 patients out of 1329 patients (3.5%) developed HCC during follow-up. There was a trend toward smaller tumor size (≤4 cm) at HCC presentation in patients with optimal surveillance versus those without (28.1% vs 60.0%, *P* = 0.084). When comparing Barcelona Clinic Liver Cancer (BCLC) tumor stage upon HCC diagnosis, patients in the optimal or suboptimal surveillance cohort were more likely to present with early BCLC stage (A or B) compared with those in the poor or no surveillance cohort, though this was not statistically significant (72.4% vs 40.0%, *P* = 0.152).

### 3.5 Persistency to adherence to HCC surveillance

**Figure 3:**
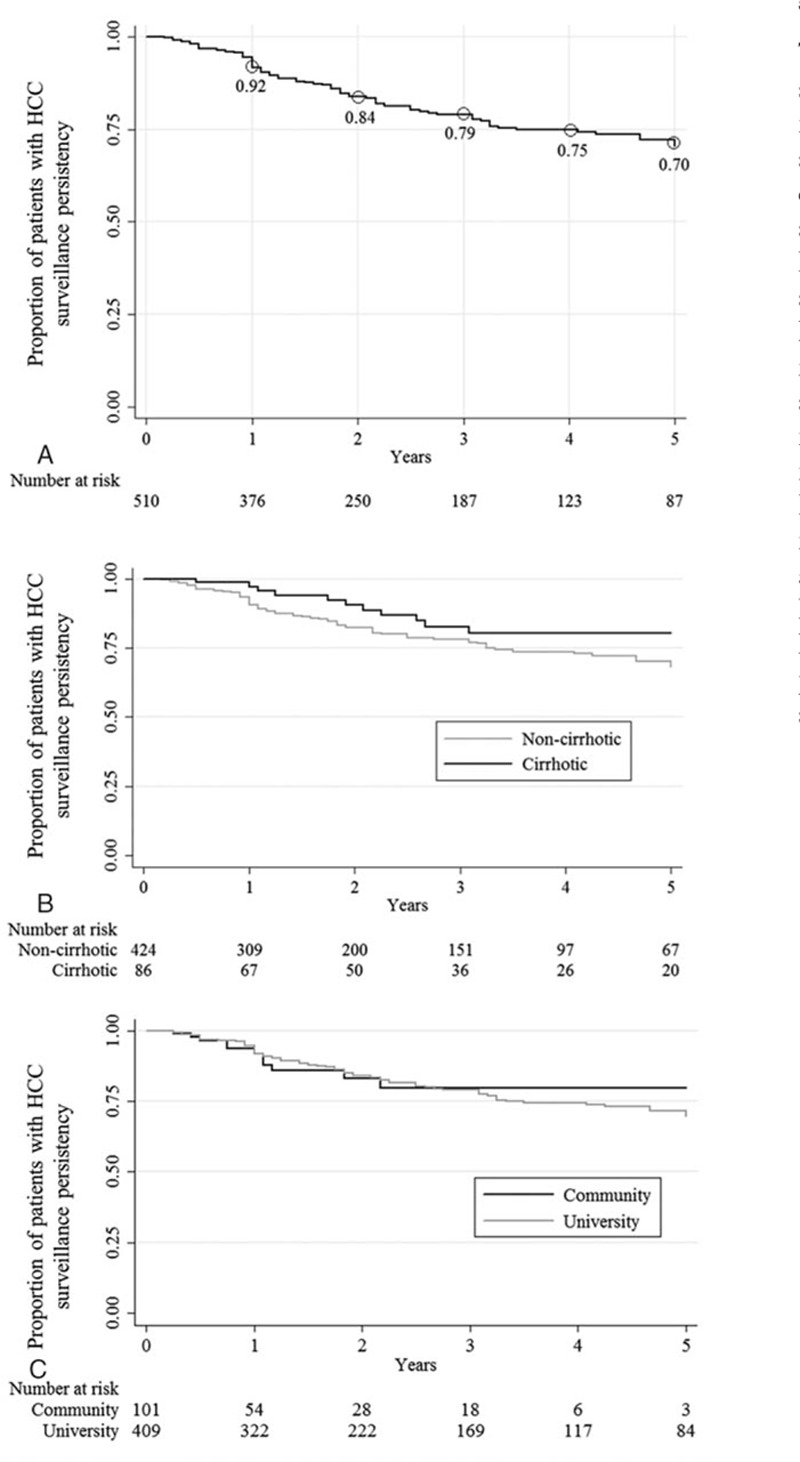
Low persistency in HCC surveillance regardless of cirrhosis status or clinical setting. A, Persistency of adherence to at least annual imaging for HCC surveillance. B, Persistency of HCC surveillance by presence of cirrhosis. C, Persistency of HCC surveillance by clinic setting. HCC = hepatocellular carcinoma.

Figure 3 describes the persistency rates of adherence to HCC surveillance in the subcohort who demonstrated consistent adherence to surveillance tests every 6 to 12 months for at least 12 months (N = 510) overall and by cirrhosis status and clinic settings. Overall, persistency to HCC surveillance adherence dropped to 84% at 2 years and 70% at 5 years (Fig. 3A) with a trend toward higher persistency in patients with cirrhosis compared with those without cirrhosis (Fig. 3B) with 5-year persistency rates of 81% and 68%, respectively (*P* < 0.010). Persistency rates to HCC surveillance adherence were similar between community and university patients (Fig. 3C).

## 4 Discussion

In this large, multicenter, retrospective cohort study of HCC surveillance patterns in consecutive patients with CHB, we found that adherence to HCC surveillance is not only poor, but also not persistent with only approximately two-thirds of those initially adherent remaining persistent after 5 years. These findings are consistent with prior studies with data on poor HCC surveillance adherence and persistence.^[20–24]^ Since CHB is a chronic disease, HCC surveillance in such patients needs to be a life-long process. These findings held in patients with and without cirrhosis and in diverse practice settings, including university-based versus community clinics found in a range of cities in the region. While there has been extensive debate on the efficacy of ultrasound versus other imaging modalities for HCC surveillance, such debate has little relevance to patient outcomes if adherence to even simple noninvasive ultrasound remains effectively low.^[11–15]^ Therefore, our data presents a strong case for further intervention to improve current adherence to HCC surveillance guidelines. The study also found that both more frequent clinical visits and university-based care were strongly associated with higher levels of adherence to HCC surveillance, following adjustments for presence of cirrhosis, age, sex, and antiviral therapy utilization. More frequent clinical visits and having clinical visits dedicated to liver care may have allowed more opportunities for both physicians and patients to focus on this important issue. Indeed, in a recent survey study, having “more important issues to manage in the clinic” was cited as one of the major reasons for not performing HCC surveillance by over half of the general practitioners.^[25]^ Therefore, the poorer surveillance results seen in community-based general gastroenterology and primary care clinics may be due to the wider range of issues to manage in these clinics.

Prior studies largely examined adherence to surveillance at single medical centers or community clinics that serve specific populations, such as Medicaid patients, or are based on survey results that are prone to recall bias by both physicians and patients.^[6,14–17]^ The current study is a real-life cohort of consecutive patients with CHB of varying disease severity (cirrhosis and noncirrhosis), from different settings (university liver clinics, community GI clinics), and across multiple cities in the San Francisco Bay Area. Therefore, results of this study can be more generalizable for a broader population of CHB patients and are highly concerning because it suggests low rates of adherence and also persistency of only 70% at 5 years even in the minority of patients who were initially more adherent to HCC surveillance. A prior study suggests that surveillance was significantly higher among patients followed in subspecialty GI clinics versus those followed in primary clinics.^[26]^ However, our results suggest that though the majority of our cohort has been followed by hepatologists and gastroenterologists (N = 1114 followed at study sites exclusively with subspecialty care), HCC surveillance remains suboptimal. These findings highlight the need for additional patient and provider education as well as a practical recall system to help improve the current practice. As the study found a strong and significant association between more frequent clinical visits and university liver clinic care with better adherence, close monitoring and access to liver specialists may help improve HCC surveillance practice. However, access to specialists and specialized liver clinics as well as surveillance tests such as ultrasound may be limited by reimbursement policies and the financial burden to patients can prove to be a major barrier for patients in many areas where these services are not universally or generally covered. Therefore, policy incentives that reduce financial barriers to screening may also improve HCC surveillance rates; an observational study finds that there was a statistically significant increase (4%) in colonoscopy rates among male Medicare beneficiaries after an Affordable Care Act policy change reduced out of pocket responsibility for the patient.^[27]^ In addition, active intervention targeting physicians and patients directly may also be helpful, as prior studies have shown that these were effective for other cancer surveillance. Interventions targeting patients, such as sending invitation letters for cervical cancer screening and reminders to patients before breast cancer screening appointments were shown to demonstrate statistically significant increases in screening participation rates.^[28,29]^ Interventions targeting physicians, such as sending cancer screening reminders and audits with feedback have been shown to increase performance in cancer screening tests.^[30,31]^

Data on the persistency of adherence to HCC surveillance are far more limited. Davila et al^[13]^ analyzed the adherence to HCC surveillance in patients with hepatitis C virus from the Veterans Affairs Health Care System and found a decline in HCC surveillance testing from the first year to the fourth year after initial follow-up. This study focused on a different population, namely veterans with hepatitis C virus infection. Furthermore, this study's definition of surveillance adherence diverged greatly from AASLD guidelines: patients were categorized as having routine tests if they had ultrasound tests done during at least 2 consecutive years in the 4 years after cirrhosis diagnosis. This definition does not account for varying frequencies of surveillance that may occur in those 2 years or afterward. Our study defines optimal surveillance based on AASLD standards and measures adherence over a time period greater than 2 years. We also studied persistency rates of adherence, which are crucial, as HCC surveillance is a life-long necessity for at-risk CHB patients.

Of note is that AASLD HCC surveillance recommendations have changed from recommending HCC surveillance 6 to 12 months to every 6 months over the study period.^[10,32]^ Such changes may contribute to suboptimal surveillance as defined by our study. However, our findings suggest that even rates of suboptimal (every 6 to 12 months) and better surveillance still remain poor.

Our study has some limitations. The study is retrospective in design and HCC adherence can be underestimated if surveillance imaging tests were performed but not available in the medical record. This underestimation is likely to be highest for university practice, a tertiary referral practice, as tests done in community facilities may not have been sent to university physicians. Second, this study is not designed to assess quality of surveillance by any individual physician or practice setting. The observed differences in HCC surveillance and adherence rates between the different practice settings are likely due, at least in part, to the differences in patient characteristics between the 2 settings in addition to the higher percentage of patients with cirrhosis at university clinics. Finally, the current study includes CHB patients largely of Asian descent, and thus our results may not be relevant to patients of other ethnicities.

## 5 Conclusion

The HCC surveillance rate and persistency of surveillance is suboptimal among CHB patients with and without cirrhosis and in university-based and community-based clinic settings. This study suggests that closer clinical follow-up with more frequent clinic visits may help improve adherence to optimal HCC surveillance. Data from this study revealed poor adherence to HCC surveillance and also poor persistence of optimal HCC surveillance. Long-term persistence to adequate adherence to HCC surveillance requires continuous vigilance and behavior reinforcement by both providers and patients. Future studies should investigate reasons for poor adherence or persistency to inform effective clinical and governmental policies that may improve HCC surveillance adherence and adherence persistency. Furthermore, future studies may investigate the efficacy of active interventions, such as provider and patient education, counseling, and quality assurance measurement in improving adherence to HCC surveillance. Studies and policies that increase adherence to HCC surveillance will be essential for increasing survival among patients with CHB and may inform effective policies in other sectors and strategies involving preventative medicine.
